# Plasma exosomal *tRNA‐derived fragments* as diagnostic biomarkers in non-small cell lung cancer

**DOI:** 10.3389/fonc.2022.1037523

**Published:** 2022-10-31

**Authors:** Baibing Zheng, Xingguo Song, Li Wang, Yue Zhang, Youyong Tang, Shiwen Wang, Lei Li, Yawen Wu, Xianrang Song, Li Xie

**Affiliations:** ^1^ Department of Clinical Laboratory, Shandong Cancer Hospital and Institute, Shandong First Medical University and Shandong Academy of Medical Sciences, Jinan, Shandong, China; ^2^ School of Medical Laboratory, Weifang Medical University, Weifang, China; ^3^ Shandong Provincial Key Laboratory of Radiation Oncology, Shandong Cancer Hospital and Institute, Shandong First Medical University and Shandong Academy of Medical Sciences, Jinan, Shandong, China

**Keywords:** exosomal tRFs, diagnosis, biomarker, plasma, NSCLC

## Abstract

**Background:**

tRNA derived small RNAs (tRFs) have recently received extensive attention; however, the effects of tRFs in exosome as biomarkers has been less studied. The objective of this study was to validate novel diagnostic exosomal tRFs with sensitivity and specificity for non-small cell lung cancer (NSCLC).

**Methods:**

Exosomes extracted from plasma of NSCLC patients and healthy individuals were identified by transmission electron microscopy (TEM), qNano and western blots. The differentially expressed tRFs were screened by high-throughput sequencing in plasma exosomes of NSCLC patients and healthy individuals, and further verified by Quantitative Real-Time PCR (qRT-PCR). To assess the diagnostic efficacy of exosomal tRFs for NSCLC, receiver operating characteristic (ROC) curves were used next.

**Results:**

The expression levels of exosomal tRF-Leu-TAA-005, tRF-Asn-GTT-010, tRF-Ala-AGC-036, tRF-Lys-CTT-049, and tRF-Trp-CCA-057 were significantly decreased in NSCLC patients and early-stage NSCLC patients compared to healthy individuals. Notably, the exepression of tRF-Leu-TAA-005, tRF-Asn-GTT-010, tRF-Ala-AGC-036, tRF-Lys-CTT-049, and tRF-Trp-CCA-057 in the exosomes were higher than the exosome depleted supernatant (EDS).

**Conclusions:**

Our results showed that the levels of exosomal tRF-Leu-TAA-005, tRF-Asn-GTT-010, tRF-Ala-AGC-036, tRF-Lys-CTT-049, and tRF-Trp-CCA-057 were significantly downregulated in NSCLC patients. This suggests that these five exosomal tRFs may be promising diagnostic biomarkers for NSCLC.

## Introduction

According to statistics, lung cancer continues to be the leading cause of death from all cancers (1). Non-small cell lung cancer (NSCLC) accounts for approximately 85% of all lung cancers and is the leading cause of high lung cancer morbidity and mortality (2). The high mortality of NSCLC is mainly because patients have missed the best treatment period and tumor metastasis. Therefore, it is urgent to study early diagnostic biomarkers for NSCLC.

Transfer RNA (tRNA) is involved in transporting specific amino acids and protein synthesis. According to recent studies, tRNA catabolism is also a significant factor leading to the production of small non-coding RNAs (tsRNA) (3). Due to the continuous development of sequencing technologies, the number of novel tsRNAs discovered is increasing. tsRNA can be roughly divided into two categories according to their cutting positions on mature tRNAs or precursor tRNAs. These two categories are referred to as tRNA halves (tiRNA) and tRFs. The former is 31 to 40 nt in length, while the latter is 14 to 30 nt in length (4). Based on the relative length of tRFs, they are divided into five main categories: i-tRF, tRF-1, tRF-2, tRF-3 and tRF-5. tRF-1 is mainly derived from the 3’ tail sequence of the precursor tRNA and contains a poly-U sequence (5). tRF-2, is a newly discovered tRFs, decomposes from the anticodon loop on tRNA (6). tRF-3, is formed on the 3’ end of mature tRNA, contains CCA part, and cuts on T-loop. tRF-5, corresponding to the 5’ end of mature tRNA, cleavage occurs in D-loop. i-tRF mainly comes from the internal region of mature tRNA (7).

As sequencing technology continues to advance, more novel tRFs have been discovered, and their role in cancer has been gradually revealed. Some studies have found that tRF belongs to short stranded non-coding RNA like microRNA, and can also bind with Argonaute proteins to form RISC complex silencing mRNA (8, 9). An example is tRFs can suppress Breast Cancer progression by binding RNA-binding protein YBX1 to displace their 30 untranslated regions (UTRs) from YBX1, thereby inhibiting the stability of oncogene transcription (10). Another example is As-tDR-007333 which promotes the progression of NSCLC by activating HSPB1 and ELK4 mediated epigenetic and transcriptional regulatory, targeting carcinogenic MED29 (11).

Numerous studies have shown that non-coding RNAs can be useful in identifying patients with various types of cancer. For instance, in a study, the researchers found that certain tRFs, such as tRF-Glu-CTC-003, tRF-GIy-CCC-007, tRF-GIy-CCC-008, tRF-Leu-CAA-003, tRF-Ser-TGA-001, and tRF-Ser-TGA-002 in plasma were found to be novel diagnostic biomarkers for early-stage Breast Cancer (12). The downregulated tDR‐7816, tDR‐5334, and tDR‐4733 in serum were considered as potential biomarkers for diagnosis of nontriple negative breast cancer (13). In addition, Londin et al. found that the level of tRF-22-BP4MJYSZH and tRF- 21-45dBNIB9b in patients with metastatic uveal melanoma (UVM) was significantly downregulated, and was related to the survival of patients (14). In conclusion, these findings suggest that tRFs might be a promising novel type of diagnostic biomarker.

Exosome is a kind of extracellular vesicle (30–150 nm diameter) secreted by a variety of cells and released into the extracellular environment, such as plasma, urine, and saliva (15). Exosomes contain a variety of lipids, proteins and RNAs that can perform a variety of biological functions and serve as transport vehicles for intercellular information exchange (16–19). Recently, there was mounting evidence indicating that exosomes could be used as diagnostic biomarkers for lung cancer (20), breast cancer (21), pancreatic cancer (22), ovarian cancer (23), and gastric cancer (24). In addition, these tRFs have also been identified in plasma and serum exosomes and stably expressed. This indicates that these tRFs also have the potential as diagnostic biomarkers, however, only a few articles have been reported. For example, plasma exosomal tRNA-ValTAC-3, tRNA-GlyTCC-5, tRNA-ValAAC-5, and tRNA-GluCTC-5 had been reported to be novel candidate biomarkers for liver cancer (25).

The purpose of this study was to explore the differential expression of plasma exosomal tRFs in NSCLC patients and healthy individuals and to assess the diagnostic efficacy of differentially expressed exosomal tRFs for NSCLC. We verified the expression levels of exosomes tRF-Leu-TAA-005, tRF-Asn-GTT-010, tRF-Ala-AGC-036, tRF-Lys-CTT-049, and tRF-Trp-CCA-057 were significantly reduced in NSCLC patients and were also found to be similarly reduced in early-stage patients with favorable diagnostic efficiency. This provides reasonable evidence for the use of exosomal tRFs as potential diagnostic biomarkers for NSCLC.

## Materials and methods

### Patients and healthy individuals

All samples were collected from Shandong Cancer Hospital from December 2019 to August 2021, including 244 untreated NSCLC patients and 250 healthy individuals. Patients with NSCLC were confirmed by combined clinical, pathological, and radiological diagnostic approaches, and the tumor stage was determined according to the eighth edition of the lung cancer TNM staging standards formulated by IASLC. Patients did not undergo any anticancer treatment or have any other endocrine, immune, or metabolic diseases. The healthy donors did not present with any other disease.This study was reviewed and approved by the Ethics Committee of the Shandong Cancer Hospital and Institute (ID: 20151202 and 2020001016). The clinical characteristics of all patients were listed in **Table 1**.

**Table 1:** Characteristics of NSCLC patients for differentially expressed plasma exosomal tRFs.

			tRF-Ala-AGC-036		tRF-Leu-TAA-005			tRF-Lys-CTT-049		tRF-Asn-GTT-010		tRF-Trp-CCA-057	
Characteristics		Cases											
			Mean ± SD	*P* value	Mean ± SD	*P* value		Mean ± SD	*P* value	Mean ± SD	*P* value	Mean ± SD	*P* value
Age (y)	<61	110	-2.878±1.509	0.338	-3.450±1.702	0.46	-9.490±1.868		0.881	-5.460±1.651	0.233	-14.358±1.576	0.786
	≥61	134	-2.698±1.415		-3.293±1.605		-9.456±1.641			-5.206±1.652		-14.305±1.456	
Gender	Male	163	-2.780±1.510	0.995	-3.361±1.710	0.858	-9.520±1.819		0.544	-5.336±1.682	0.835	-14.362±1.532	0.626
	Female	81	-2.778±1.357		-3.369±1.525		-9.375±1.588			-5.289±1.602		-14.262±1.468
Smoking	Yes	118	-2.536±1.381	0.012*	-3.124±1.599	0.028*	-9.229±1.688		0.035*	-5.081±1.581	0.028*	-14.130±1.485	0.045*
	No	126	-3.006±1.497		-3.588±1.667		-9.699±1.770			-5.544±1.693		-14.516±1.513
Drinking	Yes	79	-2.573±1.468	0.127	-3.112±1.687	0.108	-9.204±1.770		0.097	-5.143±1.719	0.249	-14.235±1.615	0.5
	No	165	-2.878±1.447		-3.485±1.620		-9.600±1.721			-5.405±1.618		-14.374±1.458
Pathology diagnosis	AC	180	-2.827±1.491	0.39	-3.424±1.694	0.407	-9.557±1.808		0.203	-5.357±1.700	0.561	-14.419±1.564	0.118
	SCC	64	-2.644±1.364		-3.194±1.511		-9.233±1.537			-5.217±1.520		-14.076±1.318
Lymph node metastasis	Yes	143	-2.610±1.493	0.031*	-3.232±1.655	0.138	-9.272±1.723		0.033*	-5.188±1.714	0.138	-14.201±1.525	0.114
	No	101	-3.018±1.379		-3.550±1.628		-9.754±1.741			-5.507±1.551		-14.511±1.473
TNM staging	0-II	100	-3.090±1.354	0.005**	-3.642±1.592	0.039*	-9.746±1.662		0.040*	-5.547±1.501	0.075	-14.577±1.528	0.033*
	III-IV	144	-2.563±1.493		-3.171±1.664		-9.281±1.779			-5.163±1.738		-14.157±1.476
Distant metastasis	Yes	89	-2.441±1.573	0.006**	-3.060±1.772	0.029*	-9.196±1.825		0.061	-4.976±1.866	0.014*	-14.262±1.579	0.602
	No	155	-2.973±1.355		-3.538±1.551		-9.630±1.681			-5.518±1.488		-14.367±1.470

AC, adenocarcinoma; SCC, squamous cell carcinoma.

*Bold value, P<0.05; **bold value, P<0.01.

### Plasma exosome isolation

Exosomes were isolated from 500 µL plasma samples by ultracentrifugation (26). In general, the centrifugation process was performed in the following order: 10 min at 3,000 g, 30 min at 10,000 g, and 2 h at 100,000 g. The separation temperature was maintained at 4°C.

### Transmission Electron Microscopy (TEM) Assay

The exosomes obtained by the above method were transferred to copper grids and fixed by adding 1% glutaraldehyde dropwise. Subsequently, 15 µL of oxalyl uranium solution was dropped in and the copper grid was stained for 5 minutes. The copper grid was washed with distilled water, and finally the purified exosomes were identified using a JEM-1200EX transmission electron microscope (jeol, Japan).

### qNano

Extracted exosome samples were examined for particle size using TRPS (qNano; Izon Science Ltd., New Zealand) according to the manufacturer’s instructions, and the data were analyzed with Izon Control Suite v.3.3.2.2000 (Izon Science Ltd.).

### Western blot analysis

Proteins extracted from exosomes and A549 cells were separated by 10% SDS-PAGE and then transferred to a PVDF membrane (Millipore, Billerica, MA, USA), followed by incubation with primary antibody for 12 h and secondary antibody for 1 h. Finally, ECL blotting detection reagents (Bio-rad, USA) were used to detect the protein bands. The antibodies and dilution ratios were used in this study are as follows: anti-GM130 (CST, 1:1000), anti-CD9 (CST, 1:500), anti-HSP70 (1:1500), and goat anti-rabbit immunoglobulin G (CST, 1:5000).

### High-throughput sequencing

Total RNA samples was pretreated to ensure the accuracy of small RNA-seq library construction. Then, DNA was synthesized and amplified using Illumina-specific primers, followed by extracting 135-160bp amplified fragments from PAGA gels and purification for library construction. Afterward, small RNA-seq was performed on Illumina NextSeq. The raw sequence data reported in this paper have been deposited in the Genome Sequence Archive (Genomics, Proteomics & Bioinformatics 2021) in National Genomics Data Center (Nucleic Acids Res 2022), China National Center for Bioinformation / Beijing Institute of Genomics, Chinese Academy of Sciences (GSA-Human: HRA003213) that are publicly accessible at https://ngdc.cncb.ac.cn/gsa-human.

### RNA Isolation and Quantitative Real-Time PCR (qRT-PCR)

Total plasma exosomal RNA was extracted using TRIzol reagent (Thermo Fisher Scientific, Carlsbad, CA, USA), followed by reverse transcription of RNA using the miRNA First Strand cDNA Synthesis Kit (AG11716). Subsequently, qRT-PCR was performed on LC480 (Roche Diagnostics, Germany) using SYBR Green Premix Pro Taq HS qPCR Kit (AG11701). ∆CT (CT^tRF^-CT^U6^) was used as a control in this study to assess the relative expression of genes. All primers were obtained from Generalbiol (Chuzhou, China), and the primer sequences were listed in **Table 2**.

**Table 2:** | Primes seqence involved.

Gene	Primer sequence(5’-3’).
U6	F:TGGAACGCTTCACGAATTTGCG R:GGAACGATACAGAGAAGATTAGC
tRF-Ala-AGC-009	F:TCCCCAGCATCTCCACCA
tRF-Glu-CTC-002	F:TCCCTGGTGGTCTAG
tRF-Ser-CGA-013	F:TCCTGTTCGTGACGCCA
tRF-Thr-CGT-009	F:GGCCCTGTAGCTCAG
tRF-Gly-GCC-037	F:TATCGATTCCCGGCCCATGC
tRF-Ala-AGC-036	F:GGGGATGTAGCTCA
tRF-Lys-CTT-049	F:GCCCAGCTAGCTCAG
tRF-iMet-CAT-014	F:GCGAAACCATCCTCTGCTACCA
tRF-Pro-CGG-008	F:GCTTCGGGTGTGAG
tRF-Val-CAC-024	F:ACCGGGCAGAAGCACCA
tRF-Thr-CGT-001	F:TATATATACCCCGTCCGTGCCTCC
tRF-Ala-AGC-046	F:GGGGGTGTAGCTCAG
tRF-Leu-TAA-005	F:ACCAGGATGGCCGAGT
tRF-Pro-TGG-011	F:TTAAAGACTTTTTCTCTGACCA
tRF-Phe-GAA-001	F:TATATACCCGGGTTTCGGCACCA
tRF-Trp-CCA-057	F:GGCCTCGTGGCGCA
tRF-Asn-GTT-010	F:CGGCTGTTAACCGAA

### Statistical analysis

The data analyses were performed with GraphPad Prism version 8.0 (GraphPad Software, San Diego, CA, United States) and SPSS 25.0 software (IBM, Ehningen, Germany). Normally distributed variables were analyzed with parametric tests; if not, they were evaluated with the Mann-Whitney test. Diagnostic efficiency was detected by ROC curve and area under the curve (AUC). All results were presented as mean ± SD (standard deviation), and *P* < 0.5 was considered statistically significant.

## Results

### The identification of isolated plasma exosome

**Figure 1:**
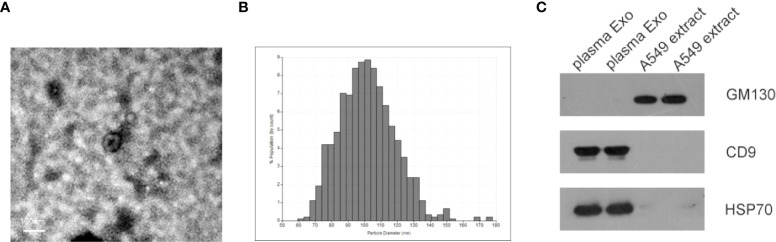
Characterization of isolated plasma exosomes. **(A)** TEM image showed representative data of exosomes with a diameter of 50-150 nm isolated from the plasma of NSCLC patients. (scale bar: 100 nm; high voltage (HV) = 80 kV). **(B)** Plasma exosome diameter detection based on qNano system, with diameters mainly distributed in the range of 70-150 nm **(C)** Western blot analysis of cell protein markers: GM130, and exosomal protein markers: CD9 and HSP70.

Identification of exosomes isolated from plasma of NSCLC patients by transmission electron microscopy (TEM), qNano. As shown in **Figures 1A, B**, most exosomes were oval vesicles with a diameter of 50-100 nm. Subsequently, high levels of exosome marker proteins CD9 and HSP70 were detected in exosome lysates, while GM130, a negative control, was only present in cell lysates (**Figure 1C**).

### tRF profiling of NSCLC patients and healthy individuals

**Figure 2:**
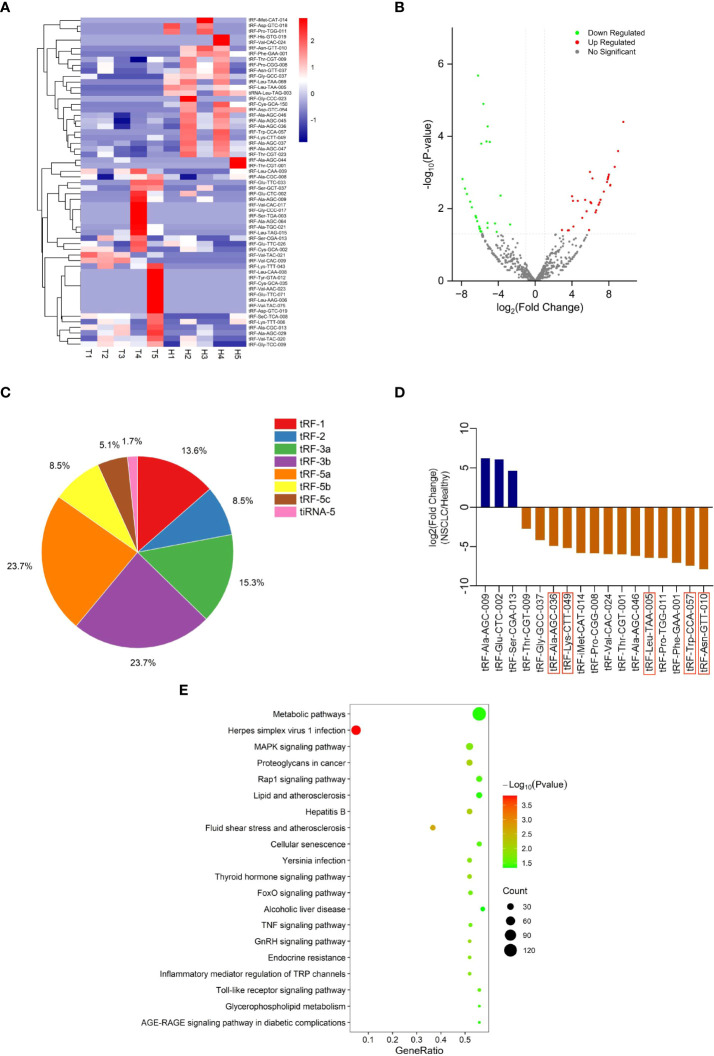
tRF profiling of NSCLC patients and healthy individuals. **(A)** Cluster heatmap and **(B)** Volcano Plot of differential expression of plasma exosomal tRFs in 5 healthy individuals and 5 patients with NSCLC. **(C)** The proportion of each subtype of differentially expressed plasma exosomal tsRNAs. **(D)** The selected differential tRFs are shown in blue (up-regulated) and brown (down-regulated), and tRF-Leu-TAA-005, tRF-Asn-GTT-010, tRF-Ala-AGC-036, tRF-Lys-CTT-049, and tRF-Trp-CCA-057 are verified to be consistent with the sequencing results. **(E)** Pathway analysis of tRF-Leu-TAA-005, tRF-Asn-GTT-010, tRF-Ala-AGC-036, tRF-Lys-CTT-049, and tRF-Trp-CCA-057.

To explore differentially expressed tRFs, we performed RNA sequencing of plasma exosomes from 5 NSCLC patients and 5 healthy individuals. A total of 680 tRFs were detected in the sequencing. For the further analysis of the differentially expressed tsRNAs among NSCLC patients and healthy individuals, all the dysregulated tsRNAs were summarized in the cluster heatmap (**Figure 2A**) and Volcano Plot (**Figure 2B**), including 32 upregulated tsRNAs and 27 downregulated tsRNAs. The pie chart showed the proportion of each subtype of differentially expressed tsRNAs (**Figure 2C**). We selected 17 of 59 tRFs for further verification in NSCLC patients and healthy individuals by qRT-PCR. It was finally confirmed that five tRFs (tRF-Leu-TAA-005, tRF-Asn-GTT-010, tRF-Ala-AGC-036, tRF-Lys-CTT-049, and tRF-Trp-CCA-057) were dysregulated in NSCLC patients and healthy individuals and consistent with the sequencing results (**Figure 2D**). To investigate the potential biological functions of these five tRFs, we performed KEGG pathway analyses of the target genes. These five tRFs were mainly involved in Metabolic, MAPK signaling, Proteoglycans in cancer, and Rap1 signaling pathways (**Figure 2E**).

### Characterization of identified five plasma exosomal tRFs

**Figure 3:**
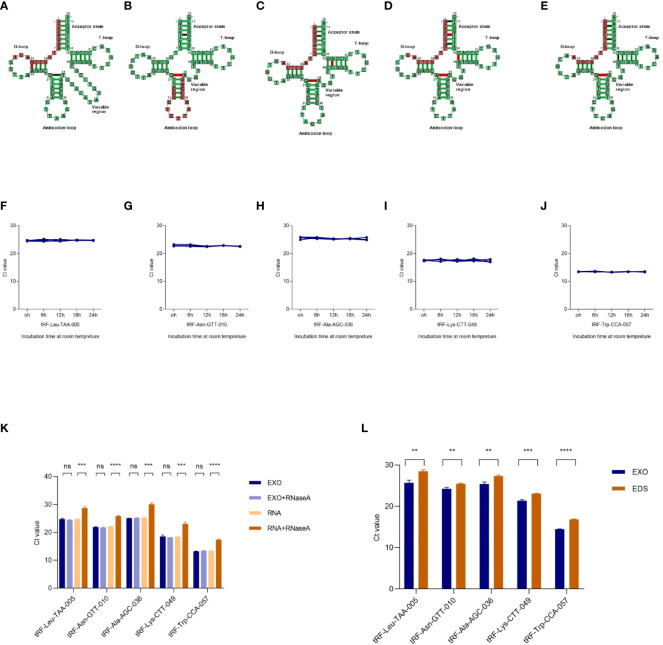
Characterization of plasma exosomal tRFs. **(A–E)** The cleavage site of tRF-Leu-TAA-005, tRF-Asn-GTT-010, tRF-Ala-AGC-036, tRF-Lys-CTT-049, and tRF-Trp-CCA-057 on their own tRNA secondary structure. The levels of **(F)** tRF-Leu-TAA-005, **(G)** tRF-Asn-GTT-010, **(H)** tRF-Ala-AGC-036, **(I)** tRF-Lys-CTT-049, and **(J)** tRF-Trp-CCA-057 when incubated at room temperature. **(K)** qRT -PCR analysis of the five tRFs in the exosomes or isolated nucleic acids treated with RNase A. **(L)** Expression levels of the five tRFs from plasma exosome (EXO) and exosome-depleted supernatant (EDS) (**p < 0.01, ***p < 0.001, ****p < 0.0001, ns, not significant).

The cleavage site of each tRF on its tRNA secondary structure predicted by tRNAdb (http://trna.bioinf.uni-leipzig.de/) was shown in **Figures 3A-E**. To analyze the characterization of exosomal tRFs, we placed the isolated exosomes at room temperature for 24h and treated them with RNase A. As shown in **Figures 3F-K**, tRFs stably expressed at room temperature and unaffected by RNase A treatment. Notably, the expression of tRF-Leu-TAA-005, tRF-Asn-GTT-010, tRF-Ala-AGC-036, tRF-Lys-CTT-049, and tRF-Trp-CCA-057 in the exosomes were higher than the exosome depleted supernatant (EDS) (**Figure 3L**).

### Decreased expression of exosomal tRF-Leu-TAA-005, tRF-Asn-GTT-010, tRF-Ala-AGC-036, tRF-Lys-CTT-049, and tRF-Trp-CCA-057 in NSCLC

**Figure 4:**
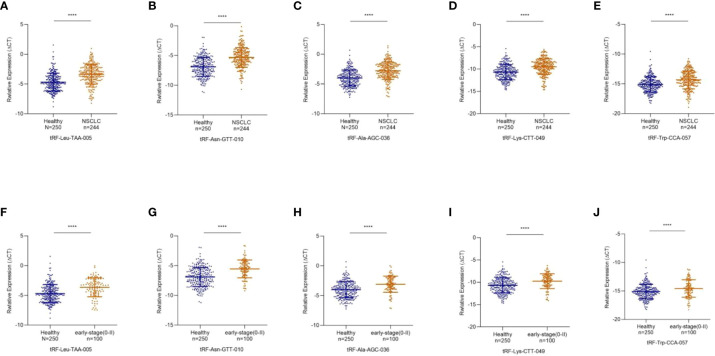
Decreased Expression of exosomal tRF-Leu-TAA-005, tRF-Asn-GTT-010, tRF-Ala-AGC-036, tRF-Lys-CTT-049, and tRF-Trp-CCA-057 in NSCLC. **(A)** tRF-Leu-TAA-005, **(B)** tRF-Asn-GTT-010, **(C)** tRF-Ala-AGC-036, **(D)** tRF-Lys-CTT-049, and **(E)** tRF-Trp-CCA-057 were significantly decreased in NSCLC patients compared with healthy controls (****p < 0.0001). The scatter plot compared the exosomal **(F)** tRF-Leu-TAA-005, **(G)** tRF-Asn-GTT-010, **(H)** tRF-Ala-AGC-036, **(I)** tRF-Lys-CTT-049 and **(J)** tRF-Trp-CCA-057 levels in the plasma of healthy individuals and early-stage NSCLC patients (****p < 0.0001).

To study differentially expressed exosomal tRFs in NSCLC, we first explored the expression levels of 17 tRFs screened in 24 NSCLC patients and 24 healthy individuals. The results revealed that the expression level of tRF-Leu-TAA-005, tRF-Asn-GTT-010, tRF-Ala-AGC-036, tRF-Lys-CTT-049, and tRF-Trp-CCA-057 were much lower in NSCLC patients than in healthy individuals, which was consistent with the results of RNA sequencing. In contrast, the other 12 tRFs did not show any significant difference. The study cohort of tRF-Leu-TAA-005, tRF-Asn-GTT-010, tRF-Ala-AGC-036, tRF-Lys-CTT-049, and tRF-Trp-CCA-057 was then expanded to 244 NSCLC patients and 250 healthy individuals (**Figures 4A-E**). The clinical characteristics of all patients included in the study were shown in **Table 1**.

Early diagnosis is essential for treating NSCLC, so we further investigated the expression levels of these five exosomal tRFs in early-stage NSCLC. As shown in **Figures 4F-J**, exosomal tRF-Leu-TAA-005, tRF-Asn-GTT-010, tRF-Ala-AGC-036, tRF-Lys-CTT-049, and tRF-Trp-CCA-057 showed significant downregulation in early-stage NSCLC patients (11 in stage 0, 61 in stage I and 28 in stage II) compared with healthy individuals.

### Exosomal tRF-Leu-TAA-005, tRF-Asn-GTT-010, tRF-Ala-AGC-036, tRF-Lys-CTT-049, and tRF-Trp-CCA-057 as biomarkers for the NSCLC diagnosis

**Figure 5:**
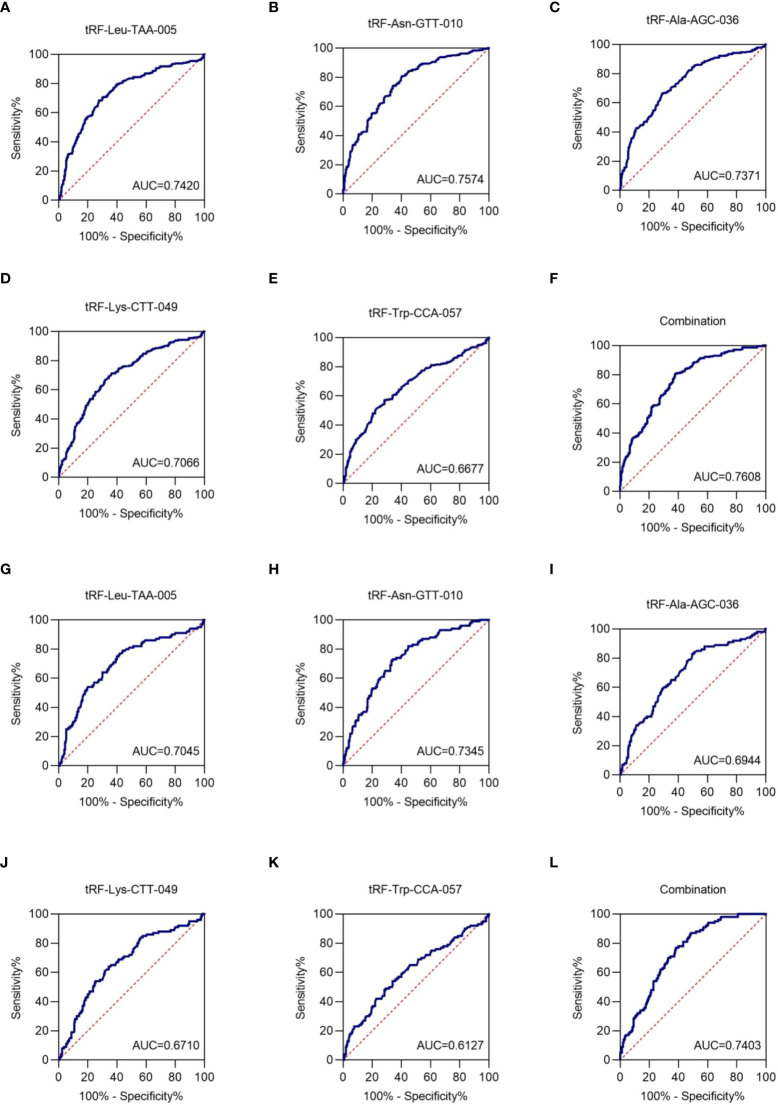
Exosomal tRF-Leu-TAA-005, tRF-Asn-GTT-010, tRF-Ala-AGC-036, tRF-Lys-CTT-049, and tRF-Trp-CCA-057 as NSCLC diagnostic biomarkers. The AUCs of **(A)** tRF-Leu-TAA-005, **(B)** tRF-Asn-GTT-010, **(C)** tRF-Ala-AGC-036, **(D)** tRF-Lys-CTT-049, **(E)** tRF-Trp-CCA-057, and **(F)** their combination in NSCLC patients relative to healthy individuals. The AUCs of **(G)** tRF-Leu-TAA-005, **(H)** tRF-Asn-GTT-010, **(I)** tRF-Ala-AGC-036, **(J)** tRF-Lys-CTT-049, **(K)** tRF-Trp-CCA-057, and **(L)** their combination in early-stage NSCLC patients relative to healthy individuals.

To assess the clinical diagnostic value of the identified exosomal tRFs, receiver operating characteristic (ROC) curves and area under the ROC curve (AUC) were used. The AUCs were 0.7420 with 68.4% sensitivity and 72.4% specificity (95% CI, 0.698-0.786), 0.7574 with 80.7% sensitivity and 59.6% specificity (95% CI, 0.698-0.786), 0.7371 with 66.4% sensitivity and 71.2% specificity (95% CI, 0.693-0.781), 0.7066 with 71.3% sensitivity and 63.6% specificity (95% CI, 0.661-0.753), and 0.6677 with 57.0% sensitivity and 71.6% specificity (95% CI, 0.620-0.716) for tRF-Leu-TAA-005, tRF-Asn-GTT-010, tRF-Ala-AGC-036, tRF-Lys-CTT-049, and tRF-Trp-CCA-057, respectively (**Figures 5A-E**). When the five tRFs were combined, the AUC increased to 0.7608, with a sensitivity of 80.7% and specificity of 62.0% (95% CI, 0.719-0.802), indicating that these five exosomal tRFs have potential as promising diagnostic biomarkers for NSCLC (**Figure 5F**).

Next, the diagnostic value of plasma exosome tRFs for early-stage NSCLC was evaluated by ROC curve. The AUC of exosomal tRF-Leu-TAA-005, tRF-Asn-GTT-010, tRF-Ala-AGC-036, tRF-Lys-CTT-049, and tRF-Trp-CCA-057 were 0.7045 with 79.0% sensitivity and 56.0% specificity (95% CI, 0.642-0.767), 0.7345 with 73.0% sensitivity and 66.0% specificity (95% CI, 0.678-0.791), 0.6944 with 83.0% sensitivity and 50.4% specificity (95% CI, 0.634-0.755), 0.6710 with 64.0% sensitivity and 65.6% specificity (95% CI, 0.608-0.734), and 0.6127 with 54.0% sensitivity and 66.0% specificity (95% CI, 0.545-0.681). Besides, the diagnostics performance of their combination possessed an AUC of up to 0.7403 with 87.0% sensitivity and 51.6% specificity (95% CI, 0.687-0.793) (**Figures 5G–L**). Therefore, we could consider tRF-Leu-TAA-005, tRF-Asn-GTT-010, tRF-Ala-AGC-036, tRF-Lys-CTT-049, and tRF-Trp-CCA-057 as potential candidates for early-stage NSCLC biomarkers.

### Plasma exosomal tRF-Ala-AGC-036 are associated with the tumor stage

**Figure 6:**
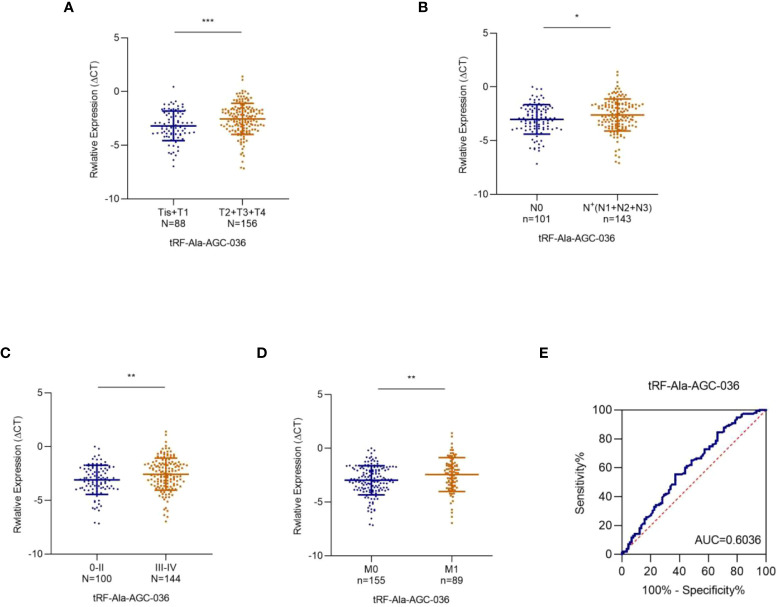
Relationship between plasma exosomal tRF-Ala-AGC-036 and tumor stage. **(A) (B)** Expression levels of exosomal tRF-Ala-AGC-036 in T and N stages NSCLC patients. **(C)** The expression level of exosomal tRF-Ala-AGC-036 of NSCLC patients with advanced stage (III-IV) was lower than that of NSCLC patients with early-stage (0- II). **(D)** Exosomal tRF-Ala-AGC-036 was downregulated in M1 stage NSCLC patients compared with M0 stage NSCLC patients. **(E)** ROC curve analysis of exosomal tRF-Ala-AGC-036 for M1 stage NSCLC patients relative to M0 stage NSCLC patients (*p < 0.05, **p < 0.01, ***p < 0.001).

Subsequently, we analyzed the relationship between the expression levels of these five tRFs and the T/N stage. As shown in **Figure 6A**, exosomal tRF-Ala-AGC-036 expression was significantly lower in the T2-T4 NSCLC patients compared to Tis-T1 NSCLC patients, whereas exosomal tRF-Ala-AGC-036 was significantly lower in the patients with lymph node positive groups compared to patients with lymph node negative (**Figure 6B**). In addition, exosomal tRF-Ala-AGC-036 accurately distinguished early (0 + II) stage and late (III + IV) stage patients (**Figure 6C**). Compared with patients with M0 stage NSCLC, exosomal tRF-Ala-AGC-036 was significantly downregulated in patients with M1 stage NSCLC (**Figure 6D**). Next, we evaluated the diagnostic efficacy of exosomal tRF-Ala-AGC-036 in NSCLC patients with distant metastasis. As shown in **Figure 6E**, the AUC of exosomal tRF-Ala-AGC-036 was 0.6036 with a sensitivity of 62.9% and a specificity of 55.5% (95% CI, 0.528-0.679). In conclusion, exosomal tRFs can be used as novel biomarkers for early diagnosis and prediction of metastasis in NSCLC.

## Discussion

Although the treatment of NSCLC is constantly updated, it still has a high recurrence rate. One of the main reasons is the lack of sensitive and specific biomarkers for early diagnosis. Therefore, the purpose of this study was to identify various types of exosomal tRFs that could be used as potential diagnostic biomarkers for NSCLC.

The lipid bilayer of exosomes prevents the degradation of exosomal cargo molecules such as DNA, mRNA, miRNA, lincRNA, rRNA, tRFs, and proteins upon release into the systemic circulation (27, 28). Exosomes can be obtained from a variety of body fluids, and their protection of the contents and their stability make them an attractive new method for detecting cancer (29, 30).

tRF, a novel small non-coding RNA derived from tRNAs (31), with the development of sequencing technologies, numerous tRFs have been discovered in exosomes (25, 32). Recently, increasingly studies have found that tRFs can be used as diagnostic biomarkers for a variety of cancers (12, 33–37), which were related to prognosis and drug resistance, and can regulate the occurrence and development of cancer (38, 39). In the current study, we screened for differentially expressed exosomal tRFs in NSCLC patients and healthy individuals by high-throughput sequencing, verified their expression levels in a large queue, and evaluated their diagnostic efficacy. After research, we found that tRF-Leu-TAA-005, tRF-Asn-GTT-010, tRF-Ala-AGC-036, tRF-Lys-CTT-049, and tRF-Trp-CCA-057 acted as novel diagnostic biomarkers for NSCLC.

First, we found that the expression level of five tRFs was significantly decreased in the plasma exosomes of NSCLC patients and early-stage NSCLC patients compared to healthy individuals, and had considerable diagnostic efficiency. More importantly, the levels of these five tRFs were significantly higher in exosomes than in plasma and were more stable. Therefore, these five exosomal tRFs may have the potential as biomarkers for the diagnosis of NSCLC.

Nevertheless, our study had some limitations. Firstly, the sample size included in this study was relatively small, and the expression of tRFs in different types of NSCLC was not analyzed. Therefore, more samples should be included in future studies. Secondly, due to the lack of clinical information of CEA in healthy individuals of our cohort, we failed to evaluate the combined diagnostic efficacy. Finally, the mechanism of these tRFs involved in the occurrence and development of NSCLC should also be further explored.

In conclusion, the current data suggested that the levels of five plasma exosomal tRFs (tRF-Leu-TAA-005, tRF-Asn-GTT-010, tRF-Ala-AGC-036, tRF-Lys-CTT-049, and tRF-Trp-CCA-057) were significantly downregulated in NSCLC patients, especially in early-stage NSCLC, which was critical for diagnosing NSCLC patients, providing novel and potential clinical diagnostic biomarkers that can accurately and rapidly distinguish.

## Data availability statement

The raw sequence data reported in this paper had been deposited in the Genome Sequence Archive (Genomics, Proteomics & Bioinformatics 2021) in National Genomics Data Center (Nucleic Acids Res 2022), China National Center for Bioinformation / Beijing Institute of Genomics, Chinese Academy of Sciences (GSA-Human: HRA003213) that are publicly accessible at https://ngdc.cncb.ac.cn/gsa-human.

## Ethics statement

This study was reviewed and approved by the Ethics Committee of the Shandong Cancer Hospital and Institute (ID: 20151002 and 2020001016). The patients/participants provided their written informed consent to participate in this study.

## Author contributions

LX was responsible for the design of the study. XS checked the data and revised the article. BZ carried out experimental research and statistical analysis, and wrote articles. LW, YZ, YT, SW, LL, YW, and XRS performed sample collection. All authors contributed to the article and approved the submitted version.

## Funding

This work was supported by Shandong Provincial Natural Science Foundation (ZR2020LZL017, ZR2019PH023), National Natural Science Foundation of China (81773237), and the Clinical Medical Science and Technology Innovation Plan of Jinan Science and Technology Bureau (202019001).

## Conflict of interest

The authors declare that the research was conducted in the absence of any commercial or financial relationships that could be construed as a potential conflict of interest.

## Publisher’s note

All claims expressed in this article are solely those of the authors and do not necessarily represent those of their affiliated organizations, or those of the publisher, the editors and the reviewers. Any product that may be evaluated in this article, or claim that may be made by its manufacturer, is not guaranteed or endorsed by the publisher.
